# Association between a *TCF4* Polymorphism and Susceptibility to Schizophrenia

**DOI:** 10.1155/2020/1216303

**Published:** 2020-03-20

**Authors:** Jia-Yang Gao, Pan Ma, Ying Li, Chun-Xia Yan, Qian Zhang, Xi-Xi Yang, Qiang Shi, Bao Zhang, Xiao-Peng Wen

**Affiliations:** ^1^College of Medicine & Forensic, Health Science Center, Xi'an Jiao Tong University, Xi'an, Shaanxi, China; ^2^Department of Radiology, The First Affiliated Hospital of Xi'an Medical University, Xi'an, Shaanxi, China; ^3^Department of Ophthalmology, First Affiliated Hospital of Xi'an Jiao Tong University, Xi'an, Shaanxi, China; ^4^Department of Thoracic Surgery, First Affiliated Hospital of Xi'an Jiao Tong University, Xi'an, Shaanxi, China

## Abstract

The basic helix-loop-helix (bHLH) transcription factor 4 (*TCF4*) had been identified as a susceptibility gene associated with schizophrenia (SCZ) by GWAS, but inconsistent results have been found in other studies. To validate these findings and to reveal the effects of different inheritance models, rs2958182, rs1261085, rs8766, and rs12966547 of the *TCF4* gene were genotyped in the Northwest Han Chinese population (448 cases and 628 controls) via a multiplex polymerase chain reaction SNPscan assay. Single SNP, genotype, and association analyses with three different models were performed. We observed genotype and allele distributions of four SNPs that showed nonsignificant associations in the Northwest Han Chinese population. However, published datasets (51,892 cases and 68,498 controls) were collected and combined with our experimental results to ascertain the association of the *TCF4* gene SNPs and SCZ, which demonstrated that rs2958182 (*P*=0.003) was a significant signal based on a systematic meta-analysis. To clarify the biological role of rs2958182, it is important to improve the understanding of the pathophysiology of SCZ.

## 1. Introduction

Schizophrenia (SCZ) is a critical and highly heritable psychotic disorder that involves genetic, environmental, and developmental factors (psychological etiologies) [1, 2]. Many adoption and twin studies have revealed that genetic factors play a notable role in SCZ, which has been estimated to explain up to 85% of the variance [3]. Although there are continuing efforts aimed at identifying the etiologies of SCZ, definite causes of the disorder have been elusive, and genetic susceptibility factors are only partly understood [4, 5]. Some SCZ-associated loci have been replicated by association studies according to genome-wide association studies (GWAS) [2, 6–8]. Nevertheless, only a few loci have been consistently associated with SCZ across multiple studies in different populations, demonstrating that the initial findings from the GWAS require replication in various independent samples, particularly in samples with different ethnic backgrounds [9, 10].

A number of interesting findings with population-wide significance have suggested that some gene variations might serve as risk markers in a subgroup of SCZ patients. Among the most confirmed genes, the basic helix-loop-helix (bHLH) transcription factor 4 (*TCF4*) is located on chromosome 18q21 and involved in normal brain development [11]. Previous studies have revealed that the *TCF4* gene is associated with many mental disorders and deficiencies, and a translocation disrupting exon 4 of *TCF4* was found associated with mental retardation and suggested a high risk for SCZ [12, 13]. In addition, the rs9960767 and rs2958182 polymorphisms of *TCF4* have been identified as susceptibility loci for SCZ, which was confirmed by a study in a European population containing 16,161 samples and a study in a Han Chinese population involving 7680 individuals, respectively [1, 14]. However, a number of studies have shown nonsignificant associations between rs2958182 and SCZ in European and Han populations [15–17]. Additionally, polymorphisms of *TCF4* have not been completely elucidated and verified in other ethnic populations.

The objective of this study was to further detect associations between the *TCF4* gene and SCZ in a Northwestern Han Chinese population. A case-control study was performed to examine the association possibility of rs1261085, rs1261084, rs8766, rs9960767, rs2958182, and rs12966547 of *TCF4* and determine the inheritance models for this study. In addition, the association between the *TCF4* gene variants and the risk of SCZ were included in a meta-analysis to clarify previous inconsistencies.

## 2. Materials and Methods

### 2.1. Subjects and Ethical Statements

A sample including 448 patients (222 males and 226 females; mean age: 36.1 ± 10.2 years) with SCZ was recruited from the Mental Health Center of First Affiliated Hospital, Xi'an Jiaotong University, from 2014 June to 2017 December. Patients were independently diagnosed by two experienced psychiatrists and met the DSM-IV criteria for SCZ, which involved an assessment of personal history, hospital records, and family-history reports. Additionally, potential patient subjects with a history of head injuries, substance-induced organic causes for psychoses, alcoholic psychosis, or psychotic disorders were excluded from the present study. Meanwhile, we recruited 628 normal controls (270 males and 358 females; mean age: 35.7 ± 9.7 years) based on medical examinations at the same hospital from 2014 June to 2017 December. The healthy controls were asked to provide detailed information about medical and family psychiatric histories for two psychiatrists. They were excluded from the study if they or their three generations relatives had a history of major mental disorders, alcohol or substance dependence, head injuries, or family history of psychiatric disorders. The healthy controls were well matched with SCZ patients in origin, age, sex, and education level. All participants were volunteers and had given their written informed consent prior to inclusion. They were longstanding residents of Shaanxi province. The study was approved by the Medical Ethics Committee of the Xi'an Jiaotong University Health Science Center (2019-916).

### 2.2. SNP Selection and Genotyping

Venous blood was collected and extracted for genomic DNA using a commercially available genomic DNA kit (Omega Bio-tek, Norcross, GA, USA). Six SNPs (rs1261085, rs1261084, rs8766, rs9960767, rs2958182, and rs12966547) of the *TCF4* gene were detected based on their likely involvement in SCZ [2, 18, 19]. As previously described, genotyping was performed by SNPscan Genotyping Assays on an ABI 7900HT Fast Real-Time PCR System (Genesky Biotechnologies Inc., Shanghai, China) according to the manufacturer's instructions [20]. Polymerase chain reaction (PCR) was used to amplify the gene fragment containing the SNP loci of interest, followed by SNPscan of genotyping frequencies. We randomly chose 5% of samples with high DNA quality and repeated the analyses to guarantee the genotyping quality. The average genotype call rate for all markers was 97.2%.

### 2.3. Statistical Analysis

The power for our sample size was calculated with the G∗Power program according to Cohen's method for statistical power [21]. The sample size of this study exhibited >80% power for the detection of significant (*P* < 0.05) associations among the genotypes, alleles, and haplotypes at an effect size index of 0.1.

The software program SHEsis or chi-square test (http://analysis.bio-x.cn) was used to assess Hardy–Weinberg equilibrium (HWE). PLINK (http://pngu.mgh.harvard.edu/purcell/plink/) software was used to analyze the allelic and genotypic association with disease [22]. Unconditional logistic regression models were used to obtain maximum-likelihood estimates of the odds ratios (ORs) and their 95% confidence intervals (CIs). Furthermore, single SNP analyses were performed using multiple inheritance models (additive, dominant, and recessive models), similar to an earlier study [23]. The data were expressed as the mean ± SD (standard deviation). Bonferroni correction was used to correct for multiple testing, and a *P* value of less than 0.05/4 was considered statistically significant.

### 2.4. Meta-Analysis

We performed a meta-analysis of previously published studies (up to May 2019) combined with our experimental data to determine the association between four polymorphisms and SCZ. Databases including PubMed, Embase, Medline, and the China National Knowledge Infrastructure (CNKI) were searched with the following keywords: “*TCF4*” and “SCZ” in combination with “genetics,” “polymorphism,” “variation,” or “association” for this study. Correlative data were independently extracted from all eligible publications by two investigators, according to strict criteria for inclusion. A third investigator discussed and arbitrated the discrepancies until consensus was reached. The following data were extracted from each article: authorship, year of publication, country of origin, ethnicity, diagnostic criteria, study type, sample size, genotyping method, genotype and allele distributions in cases and controls, and HWE of cases and controls. If information was missing from the original article, the authors were contacted and the missing data were requested.

Stata 13 software was used to conduct a meta-analysis of independent samples under dominant and recessive models. Heterogeneity between the individual studies was assessed based on the *Q* test, and *I*^2^ was detected by the chi-square test [24]. A *P* < 0.05 (*Q* test) or *I*^2^ > 50% suggested that the random effects model should be employed by the DerSimonian & Laird method [25]. Otherwise, the fixed effects model was introduced by the Mantel-Haen SCZ el method [26]. All *P* values were two-tailed, with a significance level of 0.05.

## 3. Results

### 3.1. The Experimental Data Results

The SNP rs1261084 failed to genotype in the SNPscan detection, and rs9960767 showed no polymorphic variation in our samples, which was consistent with the HapMap HCB data and previous studies. Four SNPs of *TCF4* were genotyped in 448 SCZ cases and 628 controls. There was no momentous deviation from HWE for each SNP. None of the SNPs (rs1261085, rs8766, rs2958182, and rs12966547) significantly differed in either the genotype or allele distributions, including the results of the three inheritance models (Table 1).

**Figure 1:**
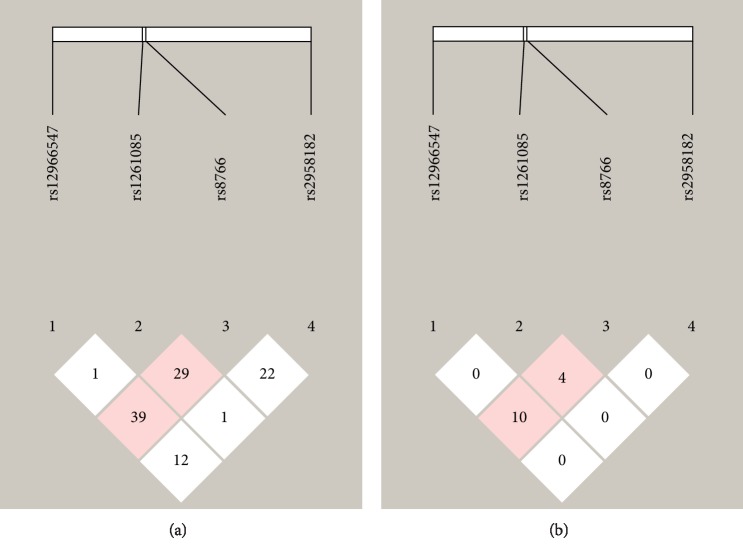
Linkage disequilibrium (LD) plots of the four SNPs of the basic helix-loop-helix (bHLH) transcription factor 4 (*TCF4*) gene. The values in the squares are the pairwise calculations of *r*^2^ (a) or D′ (b). The pink squares with “39,” for example, indicate D′ = 0.39 (i.e., low LD between a pair of SNPs). The white squares with the “0” indicate *r*^2^ = 0 (i.e., no LD between a pair of SNPs).

Linkage disequilibrium (LD) estimations for the four SNPs of the *TCF4* gene were performed. Four SNPs exhibited very low D′ and *r*^2^ values, which suggested that calculations of haplotype frequencies were not required (Figure 1). To examine whether sex influenced this association, we analyzed the data separately for the males and females based on the above mentioned results and found a nonsignificant association.

### 3.2. Pooled Studies for Case-Control Meta-analysis

**Figure 2:**
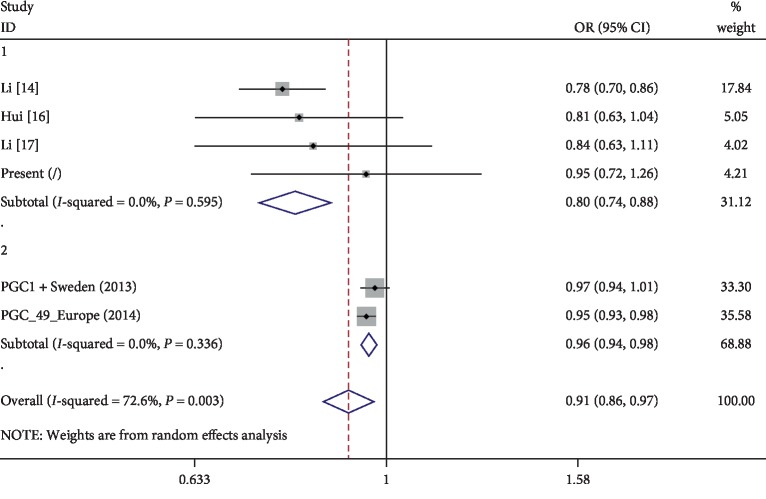
Forest plot of the *TCF4* rs2958182 polymorphism and SCZ risk in multiple populations.

We then carried out a meta-analysis of the four *TCF4* gene polymorphisms associated with SCZ. Five related studies were found in the literature search [14, 16, 17, 27, 28]. Only rs2958182 showed a significant result, which was analyzed with a total sample size of 120,390 individuals (51,892 patients and 68,498 controls). The random effects model was employed based on the heterogeneity found to be present among the six populations (*I*^2^ = 72.3%). We calculated the values of pooled OR and 95% CI for the rs2958182 variant ‘‘A” allele versus the wild-type ‘‘T” allele. There was a significant association between the *TCF4* rs2958182 polymorphism and SCZ (pooled OR = 0.914, 95% CI = 0.861–0.971, *P*=0.003) (Figure 2).

## 4. Discussion

In the present set of experimental data, the associations of rs1261085, rs8766, rs9960767, rs2958182, and rs12966547 susceptibility SNPs with SCZ were evaluated. Consistent with a previous study and the HapMap HCB data, rs9960767 showed no polymorphic variation in our samples [14]. Researchers have revealed that there was a nonsignificant difference between the SCZ and control groups for rs8766 in an Iranian population [29]. In this study, none of the four SNPs differed significantly in allele or genotype distribution in a Northwest Han Chinese population. The frequency of the rs2958182 “A” allele in this experimental study (0.108) was consistent with Li 's study (0.103) and Li's study (0.096) [14, 17]. However, Li's study (0.138) revealed that the “A” allele was slightly higher than the others, perhaps because they used chronic SCZ cases, and the location of their sampling site was far from the others [16].

We found that only the rs2958182 polymorphism was significantly associated with SCZ after the systematic meta-analysis. There are four Chinese studies included in the meta-analysis. With the exception of Tao Li's study, the other three studies of rs2958182 showed consistent results with a nonsignificant association with SCZ. It is most plausible that the contrasting findings are due to the differences in various sample sizes, geographical distributions, and detection methods across the studied populations. First, in the 49_EUR study, the association between the rs2958182 polymorphism and SCZ was nonsignificant (*P*=3.38*e* − 05) due to the Bonferroni correction to maintain the genome-wide false-positive rate at 5% [15]. Recently, Tam et al. revealed that a major limitation of the approach with GWAS was the high level of significance required to account for the multiple tests, which will result in the loss of some significant signals [30]. Second, it is real that the allele frequency and genotype distribution of the rs2958182 polymorphism showed significant differences between cases and controls in two independent studies. False negative findings (type II errors) in studies could be due to less replication and lower sample size. The reason for this may be that they missing the gene-gene interactions and gene-environment interactions [31], or because of the loss of some causative polymorphisms during linkage disequilibrium (LD) considerations [32, 33]. Taken together, due to our meta-analysis results, we obtained a strong positive signal with rs2958182, which is near to and in complete linkage disequilibrium with rs9960767, a marker considered the original positive marker at the *TCF4* gene that showed no polymorphisms in Han Chinese. The *TCF4* gene comprises 41 exons and spans 437 kb, and it is broadly expressed and plays a critical role in nervous system development [34]. As a transcription factor, the *TCF4* gene regulates the expression of other genes involved in cell differentiation, survival, and neurodevelopment [35]. Mutations of *TCF4* have been detected in some patients suspected of having Pitt-Hopkins syndrome and Angelman syndrome [36, 37]. In this study, the *TCF4* gene intronic rs2958182 polymorphism was associated with SCZ. A previous study revealed that intron variation may have important regulatory roles for several reasons: linkage with unknown causal DNA or constitute potential regulatory elements for distal genes, location in trans-splicing elements, alterations in transcription factor binding or enhancer activity, and effect on posttranslational histone modifications [38]. Therefore, identification of causality between rs2958182, *TCF4* gene regulation, and SCZ progression and outcome is crucial for understanding the mechanisms of SCZ.

There are, however, limitations regarding the interpretation of our results. First, there are considerable genetic differences between the Chinese and Caucasian populations. The subjects from different ethnicities exhibit high genetic heterogeneity related to SCZ. However, when compared within each ethnic group, there was no genetic heterogeneity for the Caucasian and Chinese Han populations. The heterogeneity was highlighted by the finding that rs9960767 was a common polymorphic variant in the Caucasian sample sets but showed no polymorphic variation in the Han Chinese sample sets. A previous study revealed that rapidly increasing sample size does not inevitably introduce a crippling degree of heterogeneity [28]. In addition, the MHC region is one of the most complex regions of the human genome and easily lends itself to population stratification. While this cannot be completely ruled out as an explanation of our positive association findings, both Chinese and Caucasian populations displayed significant signal in the meta-analysis. Second, due to a lack of access to the individual data, a lack of further adjustments for environmental risk factors and other variables in the potential gene-environment interactions might have biased the present results.

## Figures and Tables

**Table 1:** Allele and genotype frequencies of single SNP association analyses.

Markers	Allele freq (%)		HWE	*P* value^a^	OR (95% CI)	Genotype (N)			Model	OR (95% CI)	*P* value^a^
SNP ID											
rs8766	C	T				CC	CT	TT			
SCZ	49.4	50.6	0.067	0.541	1.055	110	216	115	Add	1.052 (0.890–1.244)	0.552
CTR	48.1	51.9			(0.888–1.254)	158	288	182	Dom	1.157 (0.880–1.521)	0.297
									Rec	0.989 (0.746–1.309)	0.936
rs1261085	C	T				CC	CT	TT			
SCZ	34.6	65.4	0.215	0.289	1.104	59	186	194	Add	1.100 (0.919–1.316)	0.299
CTR	32.4	67.6			(0.919–1.325)	68	268	287	Dom	1.079 (0.844–1.379)	0.545
									Rec	1.267 (0.873–1.839)	0.213
rs2958182	A	T				AA	AT	TT			
SCZ	10.8	89.2	0.641	0.731	0.953	5	85	351	Add	0.952 (0.721–1.258)	0.729
CTR	11.2	88.8			(0.723–1.255)	6	129	492	Dom	0.935 (0.692–1.261)	0.658
									Rec	1.187 (0.360–3.914)	0.778
rs12966547	G	A				GG	AG	AA			
SCZ	43.0	57.0	0.492	0.735	1.030	84	212	146	Add	1.030 (0.867–1.123)	0.738
CTR	42.3	57.7			(0.866–1.226)	115	299	212	Dom	1.038 (0.802–1.344)	0.776
									Rec	1.043 (0.763–1.424)	0.793

SCZ: schizophrenia; CTR: control; SNP: single nucleotide polymorphism; Add: additive model; Dom: dominant model; Rec: recessive model; CI: confidence interval; OR: odds ratio; *P*: *P* values of the normal chi-square statistics; HWE: Hardy–Weinberg equilibrium.

## Data Availability

The original data used to support the findings of this study are available from the corresponding author upon request.
